# Post-transplant lymphoproliferative disorder following multivisceral transplantation: Incidence, risk factors, and outcomes in an adult UK cohort

**DOI:** 10.1016/j.intf.2025.100350

**Published:** 2026-01-12

**Authors:** C. Curran, F. Kaji, F. Lopez, M. Polamreddy, A. Butler, N. Russell, A. Santarsieri, C. Rutter

**Affiliations:** a Department of Gastroenterology, Addenbrookes Hospital, Cambridge, UK; b Department of Haematology, Addenbrookes Hospital, Cambridge, UK; c Department of Transplant Surgery, Addenbrookes Hospital, Cambridge, UK

**Keywords:** PTLD, Epstein-Barr virus, Multivisceral transplant, Intestinal transplant, Alemtuzumab

## Abstract

**Background:**

Post transplant lymphoproliferative disorder (PTLD) incidence is highest in patients undergoing intestinal and multivisceral transplantation (MVT). We describe our experience of PTLD in a large cohort of adult MVT patients.

**Materials and methods:**

We performed a retrospective cohort study of 163 consecutive patients undergoing intestinal and multivisceral transplantation with alemtuzumab based induction from 2007 to 2024. Clinical data were obtained from patient records. Univariate analyses were performed to assess factors associated with PTLD development.

**Results:**

Twenty-nine patients (17.8 %) developed PTLD, of which 28/29 (96.6 %) were EBV associated. Cumulative incidence of PTLD adjusting for competing risk of death was 14.1 %, 19.7 % and 24.9 % at 1, 5, and 10 years, respectively. In those who developed PTLD median time to development of EBV viraemia was 6.6 months. Median initial EBV viral load log was 3.6. EBV serology mismatch between donor/recipient was the only variable significantly associated with PTLD development (OR 7.7, CI 2.9–20.0, P < 0.001). All patients received rituximab monotherapy initially. Of patients who had end of treatment radiological assessment (n = 23), overall response rate to rituximab was 56.5 %.

**Conclusion:**

There is a high incidence of PTLD in patients within the first year post intestinal transplant. EBV matching status is a potentially modifiable risk factor for PTLD development.

## Introduction

Intestinal and multivisceral transplantation (MVT) in adults is indicated in selected patients with intestinal failure and complications of parenteral nutrition, extensive portomesenteric vein thrombus, unresectable intrabdominal desmoid disease and widespread mesenteric arterial insufficiency causing hepatic and intestinal ischemia [1], [2], [3].

Historically associated with high morbidity and mortality [4], outcomes after intestinal transplantation are now comparable with other solid organ transplants [5]. Despite this post transplant lymphoproliferative disorder (PTLD) remains a significant complication particularly in the early stage post transplant [2], [6]. A key pathogenic driver of PTLD is infection with the Epstein–Barr virus via post-transplant viral reactivation or primary EBV infection, through the donated organ or via environmental exposure leading to uncontrolled proliferation of B cells in the context of impaired immune surveillance [7], [8], [9], [10].

The incidence of PTLD is significantly higher following MVT compared with other solid organ transplant groups [8], [11], [12]. This is thought to be explained by the large lymphoid tissue burden transplanted as part of the graft and the high burden of immunosuppressive agents required on induction such as alemtuzumab [8].

This presents a significant challenge in the post transplant care of such patients given the development of PTLD is associated with a significant decrease in patient survival [13].

While PTLD has been extensively studied in other organ groups such as liver and renal transplantation, its incidence, risk factors and clinical impact in adult recipients of intestinal containing grafts receiving modern immunosuppression strategies of lymphocyte depleting agents followed by calcineurin inhibition [4], [13] remain less well-defined. Furthermore, whilst treatment with reduction of immunosuppression (RIS) and rituximab with or without subsequent chemotherapy is established as first line treatment of PTLD in other solid organ groups there remains a lack of data on the efficacy of this in the MVT population [14], [15], [16].

Given the high associated morbidity and mortality, it is crucial to understand and mitigate the risk factors associated with developing PTLD in this cohort. We aimed to describe our experience of PTLD in a large cohort of adult patients, the risk factors associated with its development and the subsequent management and outcomes of those who developed PTLD.

## Materials and methods

### Study design

This was a retrospective cohort study of adult patients undergoing intestinal and multivisceral transplantation at a single centre in the United Kingdom. All patients undergoing intestinal and multivisceral transplantation from 1/1/2007 until 31/12/2024 were included. Data was collected from review of clinical notes, the patient electronic records, and a radiology reporting system. Patients were censored at death, development of PTLD or at the end of the follow up period on 1/11/2024.

### Variables

Data collected on recipients included demographic details (age, sex) and clinical information including, organs transplanted, CMV status, EBV status, number of alemtuzumab doses received at induction, whether splenectomy was performed at transplant, number of episodes of rejection post transplant, previous cancer diagnosis prior to transplant, previous biologic exposure prior to transplant, date of first positive EBV viraemia and EBV viral load.

Clinical and demographic details of the donors were collected including age, CMV status and EBV status. Data on patients who developed PTLD included initial treatment strategy, time to development of EBV viraemia and treatment outcome.

### Immunosuppression

All patients received induction immunosuppression with subcutaneous alemtuzumab (Campath, 30 mg) and intravenous methylprednisolone (500 mg). Until 2014 2 doses of alemtuzumab were administered; one at induction and the second on post-operative day (POD) 1.Subsequently, due to an observed increase in post-operative infections the second dose was only given if the lymphocyte count was greater than 0.05 × 109/L. Maintenance immunosuppression therapy with tacrolimus, was commenced on POD 2 (enteral Prograf or Adoport, twice daily, trough levels of 8–12 ng/ml for the first 12 months then reduced depending on variables including renal function, introduction of azathioprine or mycophenolate mofetil or rejection episodes) and intravenous methylprednisolone continued (20 mg twice daily for 7 days, 10 mg twice daily for 7 days). Oral prednisolone (20 mg daily) was commenced after POD 8 once intestinal function was adequate and weaned by 5 mg weekly to a maintenance dose of 5 mg. This was continued until the end of the first year post transplant and then stopped if there were no donor specific antibodies (DSAs) present and azathioprine or mycophenolate mofetil had been successfully introduced. Azathioprine (1 mg/kg/day) or mycophenolate mofetil (500 mg twice daily) was introduced on POD 21 if the neutrophil count allowed. This immunosuppression regime was independent of graft type or immunological risk.

### Antiviral prophylaxis

No specific antiviral prophylaxis was given for any combination of EBV status. Cytomegalovirus (CMV) prophylaxis was intravenous ganciclovir (5 mg/kg twice daily) for 1 week if either the donor or recipient were CMV immunoglobulin G (IgG) positive. This was converted to oral valganciclovir 900 mg dosed as per renal function and continued for 6–12 months. Acyclovir was given if both donor and recipient were CMV IgG negative for HSV prophylaxis.

### Management of EBV infection and PTLD

EBV viral DNA levels were monitored weekly whilst an inpatient following transplant, and at least monthly following discharge in the first 3 months and 4–6 monthly following this if negative. Following detection of EBV viraemia immunosuppression was reduced with EBV levels monitored weekly. A low threshold for cross sectional imaging including PET CT to identify radiological evidence of PTLD was adopted if viraemia was persistent. Following histological diagnosis all patients were discussed at a specialist haematologist led multidisciplinary team meeting prior to initiation of systemic anti-cancer treatment.

### Statistical analysis

Data were collected on Excel, and the statistical analysis and graph creation were performed using R studio (R version 4.4.2) and the *survival*, *ggplot2*, *dplyr*, *finalfit* and *ggplot* packages. A full list of packages used is available in the supplementary appendix. For continuous variables we judged our data to be parametric based on Q-Q plots and histograms. Chi squared testing was used to assess differences between categorical variables. The two sample T test was used to assess differences between quantitative variables. Univariate analysis was performed to determine the association between variables and the development of PTLD. Cumulative incidence of PTLD was calculated adjusting for the competing risk of death. Statistical significance was taken as p < 0.05. Where patients had missing data, these were excluded from respective analysis. No patients were lost to follow up.

## Results

### Baseline characteristics

A total of 163 consecutive patients were included in the analysis. Table 1 gives the clinical and demographic details of the total cohort. The median age at transplant was 45. 74 % were matched according to EBV status. The number of recipients receiving MVT, LSB, MMVT and SBT were 53 (33 %), 35 (21 %), 18 (11 %) and 57 (35 %) respectively.

**Table 1:** Baseline demographics and clinical characteristics of cohort.

	N = 163*
Age at transplant	45 (34, 54)
Donor Age	27 (17,44)
Sex	
- Male	88 (54%)
- Female	75 (46%)
Transplant type	
- MVT	53 (33%)
- LSB	35 (21%)
- MMVT	18 (11%)
- SBT	57 (35%)
EBV match	
- Yes	81 (74%)
- No	29 (26%)
CMV match	
- Yes	100 (65%)
- No	53 (35%)
Alemtuzumab doses at induction	
- 1	69 (58%)
- 2	49 (42%)
History of rejection	
- Yes	40 (28%)
- No	101 (72%)
Previous cancer	
- Yes	21 (18%)
- No	98 (82%)
Previous biologic use	
- Yes	16 (15%)
- No	92 (85%)
Splenectomy	
- Yes	54 (42%)
- No	74 (58%)

Median (Q1, Q3); n (%)

Table 2 gives the demographic details and baseline characteristics of the cohort stratified by those who developed PTLD and those that did not. Only EBV serology mismatch between donor/recipient was significantly different between the 2 groups (p < 0.001)

**Table 2:** Demographics details and clinical characteristics stratified by development of PTLD.

		**No PTLD** no. (%)	**Developed PTLD** no. (%)	**P value**
Age at transplant	Mean (SD)	42.9 (12.6)	44.9 (13.7)	0.445
Donor age	Mean (SD)	31.1 (14.0)	27.2 (14.4)	0.247
Sex	Female	62 (46.3)	13 (44.8)	1
	Male	72 (53.7)	16 (55.2)	
EBV match	No	14 (16.5)	15 (60.0)	**< 0.001**
	Yes	71 (83.5)	10 (40.0)	
Splenectomy	No	57 (55.3)	17 (68.0)	0.355
	Yes	46 (44.7)	8 (32.0)	
Alemtuzumab doses	1	55 (59.8)	14 (53.8)	0.751
	2	37 (40.2)	12 (46.2)	
Liver containing graft	No	58 (43.3)	17 (58.6)	0.195
	Yes	76 (56.7)	12 (41.4)	
CMV match	No	45 (36.3)	8 (27.6)	0.503
	Yes	79 (63.7)	21 (72.4)	
Rejection	No	83 (72.8)	18 (66.7)	0.69
	Yes	31 (27.2)	9 (33.3)	
Previous cancer	No	77 (83.7)	21 (77.8)	0.673
	Yes	15 (16.3)	6 (22.2)	
Previous biologic use	No	72 (86.7)	20 (80.0)	0.609
	Yes	11 (13.3)	5 (20.0)

**Table 3:** Univariate regression of clinical variables for the development of PTLD.

		**Odds Ratio (univariable)**
Age at transplant		1.01 (0.98–1.05, p = 0.443)
Donor age		0.98 (0.95–1.01, p = 0.246)
Sex	Female	-
	Male	1.06 (0.47–2.41, p = 0.888)
EBV match	No	-
	**Yes**	**0.13 (0.05–0.34, p < 0.001)**
Splenectomy	No	-
	Yes	0.58 (0.22–1.44, p = 0.253)
Doses of alemtuzumab at induction	1	-
	2	1.27 (0.52–3.07, p = 0.588)
CMV match	No	-
	Yes	1.50 (0.63–3.84, p = 0.377)
Rejection	No	-
	Yes	1.34 (0.53–3.24, p = 0.525)
Previous cancer	No	-
	Yes	1.47 (0.48–4.11, p = 0.480)
Previous biologic use	No	-
	Yes	1.64 (0.47–5.08, p = 0.408)

### Development of PTLD

**Fig. 1:**
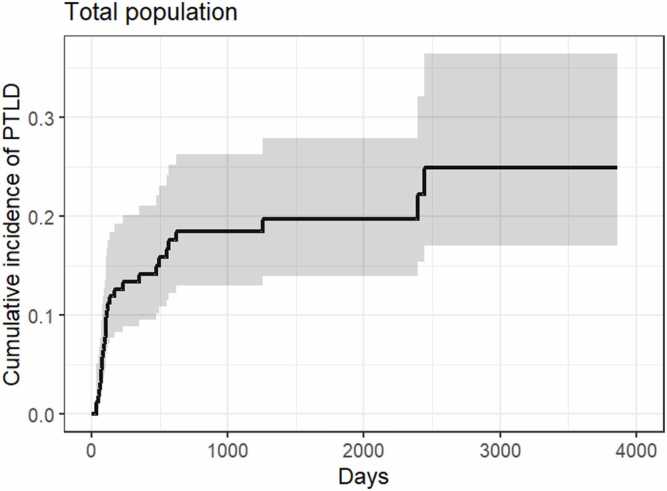
Cumulative incidence of PTLD in whole cohort adjusted for mortality.

**Fig. 2:**
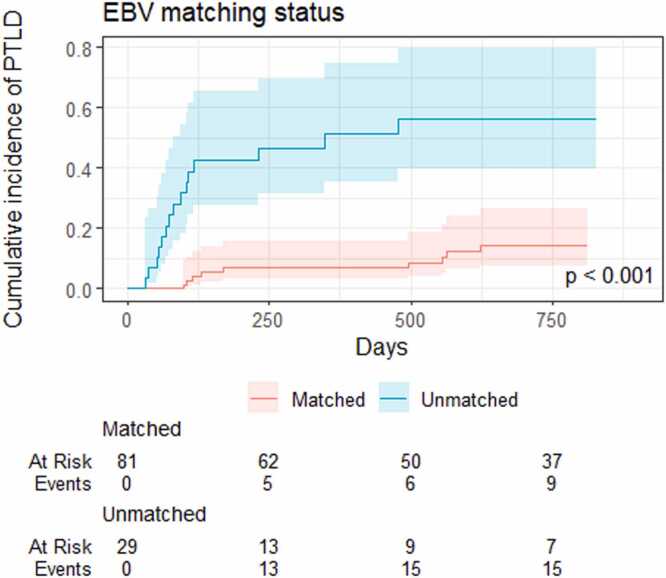
Cumulative incidence stratified by EBV matching status – matched vs unmatched.

**Fig. 3:**
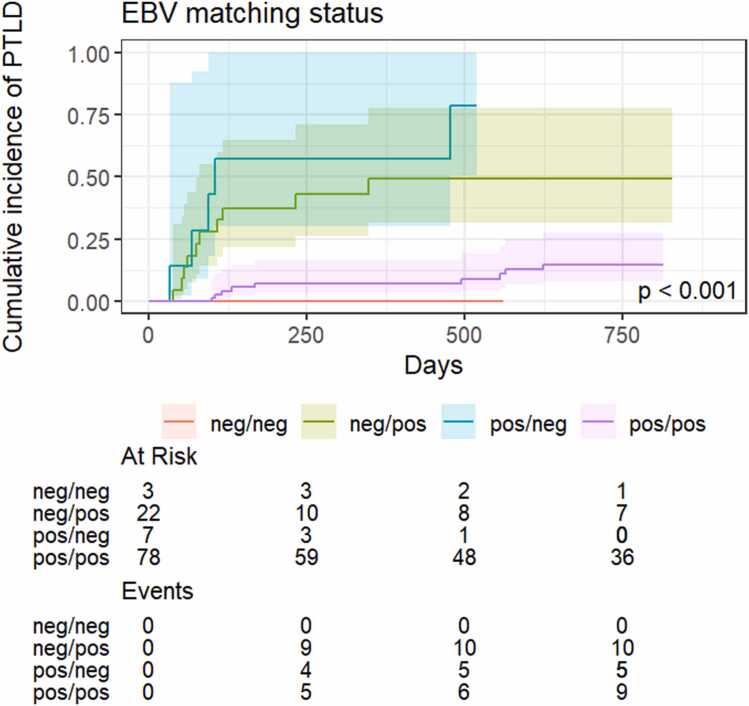
Cumulative incidence stratified by EBV matching status.

Over a mean follow up of 3.58 years (total follow up 583.1 person years) twenty-nine patients (17.8 %) developed PTLD, of which twenty eight (96.6 %) were EBV associated. The cumulative incidence of PTLD in the total cohort adjusting for competing risk of death was 14.1 %, 19.7 % and 24.9 % at one, five, and ten years respectively (Fig. 1). Cumulative incidence curves of EBV serology match/mismatch between donor/recipient are given in Fig. 2. There was no observed difference between D-/R+ and D+ /R- patients (Fig. 3). Two patients received Rituximab for persistent EBV viraemia without radiological or histological evidence of PTLD and remained well with no evidence of PTLD at the end of follow up.

### Univariate analysis

Table 2 gives the results of the univariate analysis performed. Only EBV serology mismatch between donor and recipient were significantly associated with PTLD development.

### Treatment outcomes

Treatment information was missing for two patients. Of the 27 remaining patients all received rituximab monotherapy in addition to RIS initially. Ten (37 %) achieved remission with first line rituximab with 4 patients dying during treatment. Thirteen (47 %) required second line treatment (8 patients received further rituximab, 2 patients received rituximab and chemotherapy, and 3 patients received EBV-specific cytotoxic T lymphocytes). Of these, four died due to PTLD and two died from non PTLD related causes.

Of patients who had end of treatment radiological assessment (n = 23), overall response rate to rituximab was 56.5 %. At the end of follow up 14 (52 %) were alive and in remission. Of the whole cohort eight died during treatment or directly related to PTLD while five died of non PTLD related causes. In those who developed PTLD median time to development of EBV viraemia was 6.6 months. Median initial EBV viral load log was 3.6.

## Discussion

In this paper we demonstrate the only variable associated with the development of PTLD in our cohort is EBV mismatch between donor and recipient. All patients received RIS and rituximab as first line treatment. Of those who developed PTLD at the end of follow up 14 (52 %) were alive and in remission.

The strengths of this paper include its long follow up time and large cohort of patients. Much of the published observational data on PTLD in MVT patients is in the paediatric population, we believe that this is the largest cohort of adult only patients where the risk factors for the development of PTLD and their outcomes have been studied.

It is known that there is a higher incidence of PTLD in paediatric patients compared with adults [13] however in our cohort we saw a relatively high incidence in our adult only population [17], [18]. In a large study of both adult and paediatric patients in America the cumulative incidence was 16 % at five years, with another large centre reporting a cumulative incidence of 5 % [19]. The higher incidence seen in our cohort could in part be due to the use of alemtuzumab as induction immunosuppression given it has previously been associated with an increased risk of developing PTLD compared with alternative induction agents such as ATG [20], [21]. Given all patients in this cohort underwent the same immunosuppression with alemtuzumab at induction it is not possible to confirm this association in this study. It is likely that other clinical characteristics influence this. It is notable however that receiving two doses of alemtuzumab at induction in this study was not associated with an increased risk of developing PTLD.

Furthermore, in patients undergoing kidney and pancreas transplant at our centre where alemtuzumab is used at induction the five year cumulative incidence is 11.9 % [8]. This suggests that other factors unique to intestinal transplantation such as increased donor lymphoid tissue may contribute.

Additionally, Fig. 3 shows the cumulative incidence of PTLD in individual groups based upon EBV recipient/donor status. The mismatched groups (neg/pos or pos/neg) have an earlier onset of PTLD development when compared with the matched group (pos/pos) which demonstrate a later increase in incidence. Given all patients in this cohort received the same induction regime, this may suggest unmeasured variance in maintenance immunosuppression therapy. Decisions regarding immunosuppression are often made on a case-by-case basis and are influenced by dynamic factors such as DSAs, episodes of rejection, presence of EBV and CMV viraemia and length of time from transplant.

Other factors in this study which may contribute to the higher incidence of PTLD compared with other research include characteristics related to the patient population. Due to a lack of data ethnicity was not included as a variable and may act as a confounding factor. Its is reasonable to assume however that most patients will be of White European ethnicity reflecting the demographics of the local population thus limiting extrapolation of our results to other regions that will have a more ethnically diverse population. Ultimately this highlights the necessity of multicentre collaboration when carrying out research on MVT patients given the small number of recipients worldwide and heterogenous immunosuppression regimes used [22].

The limitations of the study include its single centre retrospective nature and small absolute numbers of patients in the study. Given however the relative rarity of intestinal and multivisceral transplant compared with other solid organ transplants this is unavoidable. In previous research treatment of rejection and splenectomy were previously found to be factors associated with the development of PTLD however this was not found here [13]. This is perhaps due to this cohort consisting of only adult patients or the smaller numbers of patients limiting the power of the study.

Another factor limiting our study include missing data for several variables (Supplementary Table 1). Notably, one third of the data on EBV matching status was missing due to this not being part of the routine donor evaluation in the early years of the programme in the UK. As the missing data is not random, and the study spans an extended time period from 2007 to 2024 there could be changes in patient demographics, pre-transplant co-morbidities and physiological state, or surgical techniques that were not fully accounted for potentially confounding the results.

The use of rituximab at induction has been used to reduce the risk of de novo DSA development in the MVT cohort and in early studies appears safe and may lower the risk of acute rejection [23]. Whilst further research is needed it is biologically plausible this approach may also prove beneficial in reducing the incidence of PTLD in higher risk cohorts. Furthermore, the pre-emptive treatment of EBV viraemia prior to histological diagnosis of PTLD could be considered similar to its use in hematopoietic cell transplantation [24]. Whilst this was successfully used twice in our cohort further research is needed to validate this strategy.

## Conclusion

In conclusion given the significant increased risk of developing PTLD in patients with EBV mismatch consideration must be given to prioritising matching EBV status given the observed outcomes following the development of PTLD. Alternatively, if this is unavoidable due to an urgent need to undergo transplantation this may represent a high risk group where further management strategies are required.

## Ethical clearance

Local governance approval was sought and granted to ensure appropriate handling of patient data. Additional ethical permission for this retrospective analysis of existing data was not required

## Patients/guardian consent

Not required

## Declaration of Generative AI and AI-assisted technologies in the writing process

Not used

## Funding statement

This research did not receive any specific grant from funding agencies in the public, commercial or not-for-profit sectors.

## CRediT authorship contribution statement

**C. Curran:** Writing – original draft, Methodology, Data curation, Conceptualization. **F. Kaji:** Formal analysis, Data curation. **F. Lopez:** Writing – review & editing, Data curation. **M. Polamreddy:** Investigation, Data curation. **A. Butler:** Writing – review & editing, Formal analysis, Conceptualization. **N. Russell:** Writing – review & editing. **A. Santarsieri:** Writing – review & editing, Formal analysis, Data curation, Conceptualization. **C. Rutter:** Writing – review & editing, Supervision, Formal analysis, Data curation, Conceptualization.

## Declaration of Competing Interest

None

## Footnotes

Appendix A Supplementary data associated with this article can be found in the online version at doi:10.1016/j.intf.2025.100350.
