# Supplementary material for: Post-transplant lymphoproliferative disorder following multivisceral transplantation: Incidence, risk factors, and outcomes in an adult UK cohort
Source: Intest Fail. 2026 Jan 12;9:100350. doi: 10.1016/j.intf.2025.100350 (PMC13365921; doi:10.1016/j.intf.2025.100350)
Supplement: Supplementary file 1 — Supplementary material [file mmc1.docx]

**Supplementary Appendix**

Supplementary Table 1. Percentage of missing data

|  | Missing data (no.) |
| --- | --- |
| Age at transplant | 0 |
| Donor Age | 56 (34%) |
| Sex | 0 |
| Transplant type | 0 |
| EBV match | 53 (33%) |
| CMV match | 10 (6%) |
| Alemtuzumab doses at induction | 45 (28%) |
| History of rejection | 22 (13%) |
| Previous cancer | 44 (27%) |
| Previous biologic use | 55 (34%) |
| Splenectomy | 35 (21%) |

**Version of R and R Packages used**

R version 4.4.2

R Studio 2024.12.1 Build 563

- Arnold J (2024). _ggthemes: Extra Themes, Scales and Geoms for 'ggplot2'_. R package version 5.1.0, <https://CRAN.R-project.org/package=ggthemes>.

- Auguie B (2017). _gridExtra: Miscellaneous Functions for "Grid" Graphics_. R package version 2.3, <https://CRAN.R-project.org/package=gridExtra>.

- Chang W (2023). _extrafont: Tools for Using Fonts_. R package version 0.19, <https://CRAN.R-project.org/package=extrafont>.

- Gohel D, Skintzos P (2024). _flextable: Functions for Tabular Reporting_. R package version 0.9.7, <https://CRAN.R-project.org/package=flextable>.

- Grolemund G, Wickham H (2011). “Dates and Times Made Easy with lubridate.” _Journal of Statistical Software_, *40*(3), 1-25. <https://www.jstatsoft.org/v40/i03/>.

- Harrison E, Drake T, Pius R (2024). _finalfit: Quickly Create Elegant Regression Results Tables and Plots when Modelling_. R package version 1.0.8, <https://CRAN.R-project.org/package=finalfit>.

- Kassambara A (2023). _ggpubr: 'ggplot2' Based Publication Ready Plots_. R package version 0.6.0, <https://CRAN.R-project.org/package=ggpubr>.

- Kassambara A, Kosinski M, Biecek P (2024). _survminer: Drawing Survival Curves using 'ggplot2'_. R package version 0.5.0, <https://CRAN.R-project.org/package=survminer>.

- Makowski D, Lüdecke D, Patil I, Thériault R, Ben-Shachar M, Wiernik B (2023). “Automated Results Reporting as a Practical Tool to Improve Reproducibility and Methodological Best Practices Adoption.” _CRAN_. doi:10.32614/CRAN.package.report <https://doi.org/10.32614/CRAN.package.report>, <https://easystats.github.io/report/>.

- Müller K, Wickham H (2023). _tibble: Simple Data Frames_. R package version 3.2.1, <https://CRAN.R-project.org/package=tibble>.

- R Core Team (2024). _R: A Language and Environment for Statistical Computing_. R Foundation for Statistical Computing, Vienna, Austria. <https://www.R-project.org/>.

- Sjoberg D, Baillie M, Fruechtenicht C, Haesendonckx S, Treis T (2024). _ggsurvfit: Flexible Time-to-Event Figures_. R package version 1.1.0, <https://CRAN.R-project.org/package=ggsurvfit>.

- Sjoberg D, Whiting K, Curry M, Lavery J, Larmarange J (2021). “Reproducible Summary Tables with the gtsummary Package.” _The R Journal_, *13*, 570-580. doi:10.32614/RJ-2021-053 <https://doi.org/10.32614/RJ-2021-053>, <https://doi.org/10.32614/RJ-2021-053>.

- Sjoberg D, Yogasekaram A, de la Rua E (2025). _cardx: Extra Analysis Results Data Utilities_. R package version 0.2.4, <https://CRAN.R-project.org/package=cardx>.

- Therneau T (2024). _A Package for Survival Analysis in R_. R package version 3.7-0, <https://CRAN.R-project.org/package=survival>. Terry M. Therneau, Patricia M. Grambsch (2000). _Modeling Survival Data: Extending the Cox Model_. Springer, New York. ISBN 0-387-98784-3.

- Wickham H (2016). _ggplot2: Elegant Graphics for Data Analysis_. Springer-Verlag New York. ISBN 978-3-319-24277-4, <https://ggplot2.tidyverse.org>.

- Wickham H (2023). _forcats: Tools for Working with Categorical Variables (Factors)_. R package version 1.0.0, <https://CRAN.R-project.org/package=forcats>.

- Wickham H (2023). _stringr: Simple, Consistent Wrappers for Common String Operations_. R package version 1.5.1, <https://CRAN.R-project.org/package=stringr>.

- Wickham H, Averick M, Bryan J, Chang W, McGowan LD, François R, Grolemund G, Hayes A, Henry L, Hester J, Kuhn M, Pedersen TL, Miller E, Bache SM, Müller K, Ooms J, Robinson D, Seidel DP, Spinu V, Takahashi K, Vaughan D, Wilke C, Woo K, Yutani H (2019). “Welcome to the tidyverse.” _Journal of Open Source Software_, *4*(43), 1686. doi:10.21105/joss.01686 <https://doi.org/10.21105/joss.01686>.

- Wickham H, François R, Henry L, Müller K, Vaughan D (2023). _dplyr: A Grammar of Data Manipulation_. R package version 1.1.4, <https://CRAN.R-project.org/package=dplyr>.

- Wickham H, Henry L (2023). _purrr: Functional Programming Tools_. R package version 1.0.2, <https://CRAN.R-project.org/package=purrr>.

- Wickham H, Hester J, Bryan J (2024). _readr: Read Rectangular Text Data_. R package version 2.1.5, <https://CRAN.R-project.org/package=readr>.

- Wickham H, Vaughan D, Girlich M (2024). _tidyr: Tidy Messy Data_. R package version 1.3.1, <https://CRAN.R-project.org/package=tidyr>.
